# Actigraphic Measures of Sleep Quality Associated With Ambulatory Cognitive Performance in Older Adults

**DOI:** 10.1093/geroni/igaa057.1924

**Published:** 2020-12-16

**Authors:** Orfeu Buxton, Ruixue Zhaoyang, June Jiao, Rachel Zimmerman, Martin Sliwinski, Carol Derby

**Affiliations:** 1 Pennsylvania State University, University Park, Pennsylvania, United States; 2 The Pennsylvania State University, University Park, Pennsylvania, United States; 3 Penn State University, University Park, Pennsylvania, United States; 4 Albert Einstein College of Medicine, Bronx, New York, United States

## Abstract

Few longitudinal studies link objectively assessed sleep and cognitive performance in ecologically-valid environments. Participants enrolled in the community-based Einstein Aging Study cohort (n=224, M[age]=77.17). Wake after sleep onset (WASO) was associated with worse cognitive performance with and without MCI (2-week smartphone-based EMA, six assessments/day). Models include age, gender, ethnicity, education, learning effects, sleep duration, WASO*MCI interaction (p=0.015 for Symbol Match; p=0.002 for Color Shape). Associations were stronger among those with MCI, thirty minutes more nightly WASO predicted a 500ms longer Symbol Match response time (p<0.0001), 5.05% higher Color Dot error proportion (p=0.002), 0.184 points lower Color Shape accuracy (p<0.0001). In those without MCI, WASO was associated with worse cognition: thirty minutes more nightly WASO predicted +166.7ms Symbol Match response time (p=0.03) and -0.06 points Color Shape accuracy (p=0.013). Actigraphic sleep quality associated with ambulatory cognitive performance (and worse with MCI status) suggests targets for prevention/mitigation of cognitive decline. Part of a symposium sponsored by the Sleep, Circadian Rhythms and Aging Interest Group.

